# Supplementary material for: Improved Motion Artifact Correction in fNIRS Data by Combining Wavelet and Correlation-Based Signal Improvement
Source: Sensors (Basel). 2023 Apr 14;23(8):3979. doi: 10.3390/s23083979 (PMC10146128; doi:10.3390/s23083979)
Supplement: Supplementary file 1 [file sensors-23-03979-s001.zip › sensors-2254945-supplementary.pdf]

Supplemental Table S1. The processing duration for each MA correction method. The durations required for the processing of 41.5 minutes of data recorded in 8 channels (8 HbO and 8 HbR) sampled at 10 Hz. The analysis was run with HOMER3 on a PC with a Windows 10 operating system and an intel® core™ i5-7500 @3.4 GHz processor.

Method	Processing time (Sec)
Uncorrected	-
RLOESS	690 ±6
WCBSI	65 ±0.2
Wavelet	62 ±0.2
splineSG	21 ±0.3
PCA	17 ±0.5
spline	17 ±0.4
tPCA	16 ±0.5
CBSI	16 ±0.5
